# Complex Coordination of Cell Plasticity by a PGC-1α-controlled Transcriptional Network in Skeletal Muscle

**DOI:** 10.3389/fphys.2015.00325

**Published:** 2015-11-09

**Authors:** Barbara Kupr, Christoph Handschin

**Affiliations:** Biozentrum, University of BaselBasel, Switzerland

**Keywords:** skeletal muscle, transcriptional regulation, PGC-1α, exercise, metabolism, co-regulator

## Abstract

Skeletal muscle cells exhibit an enormous plastic capacity in order to adapt to external stimuli. Even though our overall understanding of the molecular mechanisms that underlie phenotypic changes in skeletal muscle cells remains poor, several factors involved in the regulation and coordination of relevant transcriptional programs have been identified in recent years. For example, the peroxisome proliferator-activated receptor γ coactivator-1α (PGC-1α) is a central regulatory nexus in the adaptation of muscle to endurance training. Intriguingly, PGC-1α integrates numerous signaling pathways and translates their activity into various transcriptional programs. This selectivity is in part controlled by differential expression of PGC-1α variants and post-translational modifications of the PGC-1α protein. PGC-1α-controlled activation of transcriptional networks subsequently enables a spatio-temporal specification and hence allows a complex coordination of changes in metabolic and contractile properties, protein synthesis and degradation rates and other features of trained muscle. In this review, we discuss recent advances in our understanding of PGC-1α-regulated skeletal muscle cell plasticity in health and disease.

## Introduction

Cell plasticity is often controlled by complex transcriptional networks. While traditionally, much of the research on such networks was focused on transcription factors, the important role of co-regulators has been increasingly appreciated in recent years (Dasgupta et al., 2014; Mouchiroud et al., 2014). Co-regulator proteins have no intrinsic DNA-binding domain and thus rely on transcription factors to be recruited to regulatory elements. The ability of co-regulators to bind to various partners enables the regulation of broad, complex transcriptional programs. The peroxisome proliferator-activated receptor γ (PPARγ) coactivator-1α (PGC-1α) is a prototypical member of this class of proteins. Initially discovered in a screen comparing white and brown adipose tissue, expression of PGC-1α has subsequently been documented in all tissues with a high energetic demand, including brain, kidney, skeletal, and cardiac muscle, liver, pancreas, or the retina (Martínez-Redondo et al., 2015). The core function of PGC-1α centers on the induction of mitochondrial biogenesis and oxidative metabolism. PGC-1α likewise controls highly tissue-specific programs, such as hepatic gluconeogenesis or mitochondrial uncoupling in brown adipose tissue. Since the phenotype of global PGC-1α transgenic and knockout mice is complex (Lin et al., 2004; Liang et al., 2009), many insights into organ-specific function and regulation of PGC-1α, including those discussed in this review unless otherwise stated, have mostly been obtained from murine tissue-specific gain- and loss-of-function models (Handschin et al., 2005). In skeletal muscle, specific overexpression of PGC-1α at physiological levels is sufficient to induce an endurance-trained phenotype (Lin et al., 2002) while super-physiological overexpression promotes fiber damage and impaired muscle function (Lin et al., 2002; Miura et al., 2006). Inversely, ablation of PGC-1α gene expression in this tissue promotes several typical signs of pathological inactivity, including a local and systemic chronic inflammation (Handschin et al., 2007a,b). A reduction in PGC-1α gene expression in human skeletal muscle has been associated with insulin resistance and type 2 diabetes, at least in some patient cohorts (Patti et al., 2003). Of note, mice with a heterozygous deletion of PGC-1α in skeletal muscle also exhibit a dysregulation of glucose homeostasis (Handschin et al., 2007b).

PGC-1α integrates the activity of the major signaling pathways that are important in a contracting muscle fiber and accordingly, PGC-1α transcript and protein levels are elevated after a training bout (Pérez-Schindler and Handschin, 2013). As consequence, PGC-1α then controls the biological program encompassing all plastic changes of the muscle cell to endurance exercise. For example, mice with elevated muscle PGC-1α levels exhibit a higher number of mitochondria, a switch toward oxidative, slow-twitch muscle fiber types, altered substrate synthesis and metabolism, and a reduction in muscle protein breakdown (Handschin, 2010). Importantly, the effects of PGC-1α extend beyond muscle cells: elevation of PGC-1α in muscle leads to higher tissue vascularization (Arany et al., 2008) and a remodeling of the post- and presynaptic side of the neuromuscular junction (Handschin et al., 2007c; Arnold et al., 2014). Furthermore, PGC-1α-regulated endocrine mediators, members of the so-called myokine protein family, promote beige fat cell differentiation and activation in white adipose tissue or neurogenesis in the hippocampus and thereby dramatically extend the reach of muscle PGC-1α (Schnyder and Handschin, 2015). Surprisingly, while overexpression of PGC-1α is sufficient to induce an endurance-trained muscle phenotype, several aspects of training adaptation seem to be retained even in mice with a global or muscle-specific knockout of this coactivator, respectively (Leick et al., 2008; Rowe et al., 2012). Discrepancies in such studies however indicate that the specific animal model, training type, timing, intensity, and other aspects of the exercise protocol are important for the assessment of the requirement of PGC-1α in exercise adaptation (Geng et al., 2010). Furthermore, these studies imply a redundant regulation of these evolutionarily extremely important plastic changes in skeletal muscle in which PGC-1α can be partially replaced by other, so far unknown regulators. Thus, in a physiological setting, PGC-1α controls a highly complex transcriptional network that requires spatial and temporal specification, coordination of anabolic and catabolic pathways as well as precise activation and termination. In this mini review, we highlight some of the recent mechanistic findings that contribute to the ability of PGC-1α to regulate transcription in such a broad and precise manner.

## Integration of contraction-induced signaling pathways by PGC-1α in skeletal muscle

**Figure 1:**
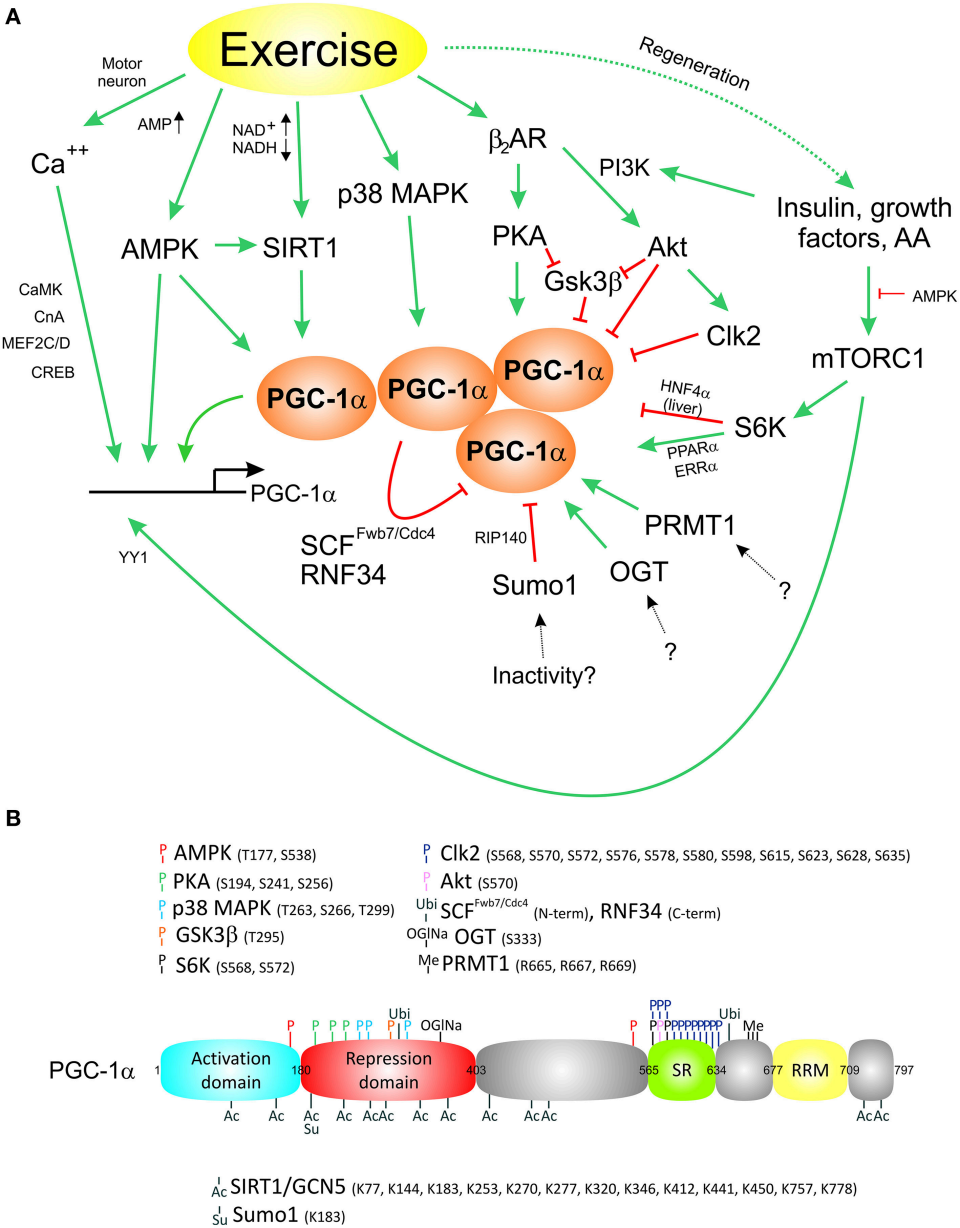
**Complex activation of the transcriptional co-activator PGC-1α by different signaling pathways**. **(A)** Exercise triggers a complex transcriptional activation as well as various posttranslational modifications to control PGC-1α levels and activity. **(B)**, Posttranslational modifications of the PGC-1α protein. AA, amino acids; Ac, acetylation; Akt, protein kinase B; AMPK, AMP-activated protein kinase; β2AR, β2 adrenergic receptor; CaMK, Ca^2+^/calmodulin-dependent protein kinase; CnA, calcineurin A; Clk2, Cdc2-like kinase 2; CREB, cAMP response element binding protein; ERRα, estrogen-related receptor α; Gsk3β, glycogen synthase kinase 3 beta; HNF4 α, hepatic nuclear factor 4 α; MEF2C/2D, myocyte enhancer factors 2C/D; Me, methylation; mTORC1, mammalian target of rapamycin complex 1; OGlNa, O-linked β-N-acetylglucosamination; OGT, O-linked β-N-acetylglucosamine transferase; p38 MAPK, p38 mitogen-activated protein kinase; PI3K, phosphoinositide 3 kinase; PKA, protein kinase A; P, phosphorylation; PPARα, peroxisome proliferator-activated receptor α; PRMT1, protein arginine methyltransferase 1; RIP140, receptor interacting protein 140; RNF34, ring finger protein 34; S6K, S6-kinase; SCF^*Fwb*7∕*Cdc*4^, SCF ubiquitin ligase complex subunit Fwb7/Cdc4; SIRT1, sirtuin-1; Su, sumoylation; Sumo1, small ubiquitin related modifier 1; Ubi, ubiquitination; YY1, yin yang 1.

Muscle fiber contraction is linked to activation by the motor neuron, mechanical stress, relative tissue physoxia, an altered neuroendocrine milieu, changes in metabolic demand and other stimuli that engage various signaling pathways. All of these signals converge on PGC-1α and promote a transcriptional induction of the gene or induce posttranslational modifications (PTM) of the protein (Figure 1A), including PGC-1α protein phosphorylation by various kinases on different phosphorylation sites, acetylation, methylation, sumoylation, ubiquitination, and acetylglucosamination (Figure 1B; Fernandez-Marcos and Auwerx, 2011). The effects of most of these modifications on PGC-1α function are still poorly understood. Some of the PTMs can alter the stability of the PGC-1α protein or modulate the interaction with transcription factors or other co-regulators. For example, phosphorylation by the p38 mitogen-activated protein kinase (p38 MAPK) results in a prolongation of the normally very short half-life of the PGC-1α protein of ~2.5 h (Puigserver et al., 2001), at least in part by preventing ubiquitination of PGC-1α and therefore stabilizing the protein (Olson et al., 2008). The AMP-dependent protein kinase (AMPK) likewise phosphorylates the PGC-1α protein in addition to its positive effect on PGC-1α gene transcription and predominantly triggers catabolic pathways to rectify a relative energy deficit, e.g., in exercise (Jager et al., 2007). Such a temporal specification is extremely important to avoid futile cycles of PGC-1α-controlled anabolic and catabolic pathways, e.g., fatty acid β-oxidation and *de novo* lipogenesis (Summermatter et al., 2010). Moreover, PTMs could also determine spatial differentiation of PGC-1α function. For example, the interaction between PGC-1α and the GA-binding protein (GABP, also called nuclear respiratory factor 2 or NRF2) not only requires the presence of host cell factor (HCF) as an additional adaptor protein, but also specific phosphorylation events both on PGC-1α as well as the GABPB1 subunit of the GABP complex (Handschin et al., 2007c). These PTMs can be triggered by motor neuron-evoked neuregulin stimulation of the muscle fiber and thereby control a specific transcriptional activation of post-synaptic neuromuscular junction genes by PGC-1α and GABP exclusively in sub-synaptic nuclei (Handschin et al., 2007c). Thus, in addition to the modulation of protein stability, PTMs might alter the activity and stability of PGC-1α as well as the ability to interact with transcription factors and thereby regulate specific transcriptional programs (Handschin and Spiegelman, 2006). For most modifications of the PGC-1α protein, a “PTM code” (Lonard and O'malley, 2007) that determines transcription factor interaction specificity has not been elucidated. A prototypical example for a PGC-1α PTM code however is provided by the S6 kinase (S6K)-mediated phosphorylation that selectively retains the ability of PGC-1α to boost fatty acid oxidation and mitochondrial function while attenuating its effect on gluconeogenesis in the liver (Lustig et al., 2011). Mechanistically, these PTMs reduce the interaction between PGC-1α and the hepatic nuclear factor 4 α (HNF4α), but not co-activation of the estrogen-related receptor α (ERRα) or PPARα. In addition to binding to transcription factors, PTMs of the PGC-1α protein can also affect the interaction with other co-regulators. For example, the co-repressors p160 myb binding protein (p160MBP) and receptor interacting protein 140 (RIP140) are recruited to PGC-1α in a PTM-dependent manner: p160MBP inhibits the ability of PGC-1α to regulate mitochondrial gene expression in the absence of p38 MAPK-mediated phosphorylation (Fan et al., 2004) while RIP140 associates with and represses sumoylated PGC-1α (Rytinki and Palvimo, 2009). Other co-repressors reduce PGC-1α activity by competing for binding to transcription factors, for example modulation of ERRα co-activation by the nuclear receptor co-repressor 1 (NCoR1; Pérez-Schindler et al., 2012) or of the glucocorticoid receptor by the small heterodimer partner (SHP; Borgius et al., 2002). PTM-dependent binding events are also observed for co-activators as exemplified by the interaction of PGC-1α with the Mediator 1 (MED1) subunit of the TRAP/DRIP/mediator complex that is disrupted after phosphorylation of PGC-1α by the Cdc2-like kinase 2 (Clk2; Tabata et al., 2014). Ubiquitination and subsequent proteasomal degradation of the PGC-1α protein form a negative feedback loop to ensure timely termination of the PGC-1α response (Sano et al., 2007). This process might be triggered by PGC-1α protein self-aggregation upon reaching a critical threshold (Sano et al., 2007). Thus, PGC-1α serves as recipient of a multitude of PTMs, thereby integrates the activity of the respective signaling pathways and subsequently triggers a transcriptional response that is adapted to the specific cellular context.

## Regulatory and functional diversity based on the PGC-1α gene structure and transcript variants

PGC-1α gene expression is rapidly and robustly increased in response to external stimuli that increase the energy demand such as cold in brown fat, fasting in the liver, or contraction in skeletal muscle (Lin et al., 2005). The cAMP response element binding protein (CREB) and the activating transcription factor-2 (ATF-2), both of which bind to cAMP response elements (CRE), are common regulators of PGC-1α transcription in most tissues. In addition, tissue-specific transcription factors provide an additional layer of control, e.g., myocyte enhancer factors 2C/D (MEF2C and -2D) in muscle cells (Pérez-Schindler and Handschin, 2013). A positive autoregulatory loop of MEF2C/D co-activation by PGC-1α on its own promoter furthermore contributes to adequate and controlled induction of PGC-1α transcription in this tissue (Handschin et al., 2003). Intriguingly, this theme of using cross- and autoregulatory loops, thus forming biological switches, is also observed in early downstream target gene regulation, for example in the induction of ERRα and GABPA by PGC-1α (Mootha et al., 2004).

PGC-1α transcription can be initiated from three start sites on two alternative promoters. Moreover, alternative RNA processing further increases the number of PGC-1α transcripts and, as a consequence, protein variants (Martínez-Redondo et al., 2015). Even though the regulatory elements of the two promoters have not yet been studied in detail, the proximal promoter seems to provide a more robust basal expression while the distal promoter that is approximately 13 kb upstream exhibits a higher dynamic range in gene expression, at least in skeletal muscle (Martínez-Redondo et al., 2015). It is still unclear whether alternative promoter usage is closely linked to the transcription of PGC-1α isoforms. Moreover, the functional consequence of the selective expression of most PGC-1α transcript variants is unknown. Surprisingly however, the PGC-1α4 variant seems to regulate a highly distinct transcriptional program with very little overlap compared to that of the other variants (Ruas et al., 2012). PGC-1α 4 contributes to skeletal muscle adaptation to resistance training and the ensuing fiber hypertrophy (Ruas et al., 2012), at least in certain contexts (Pérez-Schindler et al., 2013), diametrically opposite to the endurance exercise-like phenotype triggered by the other PGC-1α isoforms. In humans however, some studies questioned an exclusive correlation between PGC-1α4 expression and resistance training (Lundberg et al., 2014) warranting further studies. In any case however, the gene structure and transcript processing of PGC-1α thus provide an additional layer of regulatory and functional specification (Handschin and Spiegelman, 2006).

## PGC-1α-controlled transcriptional network regulation allows context-dependent control and specification

**Figure 2:**
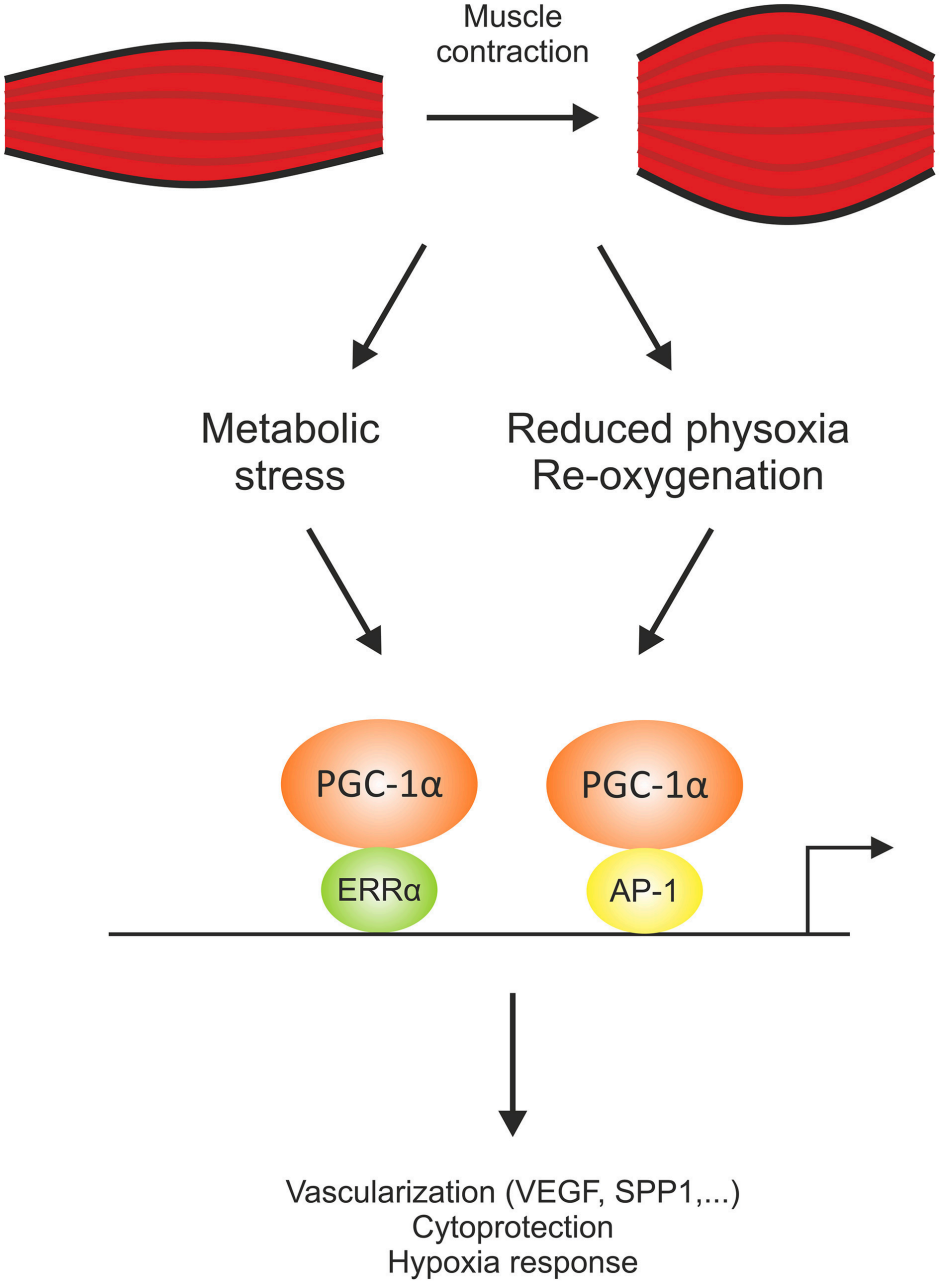
**PGC-1α engages different transcription factors to robustly regulate biological programs**. Co-activation of the estrogen-related receptor α (ERRα) and the activator protein-1 transcription factor complex (AP-1) allow a differential regulation of the gene program for vascularization, cytoprotection, and hypoxia response triggered by different stimuli, e.g., a change in the metabolic demand and the ensuing angiogenesis to improve substrate availability or neovascularization as a response to a contraction-mediated reduction in tissue physoxia and an ensuing re-oxygenation for adequate oxygen supply. SPP1, secreted phosphoprotein 1; VEGF, vascular endothelial growth factor.

Originally, PGC-1α has been discovered as a coactivator of PPARγ and was hence named accordingly (Puigserver et al., 1998). It however became clear that PGC-1α not only interacts with this, but also a number of other nuclear receptors and non-nuclear receptor transcription factors. Intriguingly, these interactions are mediated by different structural domains within the PGC-1α protein, with a more N-terminal preference for nuclear receptor co-activation while others, for example MEF2 or forkhead box protein 1 (Foxo1) bind PGC-1α closer to the C-terminus (Lin et al., 2005). Therefore, the functional specificity of PGC-1α isoforms that lack certain domains of the full-length protein could stem from the specific ablation and enhancement of binding to transcription factor partners (Handschin and Spiegelman, 2006; Martínez-Redondo et al., 2015). Despite the identification of various interaction partners, PGC-1α seems to have a special relationship with ERRα, at least in the regulation of mitochondrial genes (Mootha et al., 2004). ERRα is an orphan nuclear receptor that is mainly activated by co-activator binding in a ligand-independent manner (Kallen et al., 2004). Moreover, PGC-1α rapidly induces the transcription of ERRα as an early response target gene (Mootha et al., 2004). It thus is not surprising that pharmacological inhibition of the interaction between PGC-1α and ERRα or knockdown of ERRα has a potent effect on many PGC-1α target genes in muscle cells (Mootha et al., 2004; Schreiber et al., 2004). A global analysis of PGC-1α recruitment to regulatory elements in the mouse genome combined with an expression analysis however revealed a so-far largely underestimated number of putative transcription factor partners beyond ERRα to be involved in PGC-1α-mediated target gene regulation in muscle cells (Baresic et al., 2014). Furthermore, transcription factor motif activity response analysis not only predicts ERRα to be involved in the regulation of primary, but also secondary PGC-1α target genes, thus both in the co-activated state but also working without PGC-1α in this context. These data indicate a much higher complexity of transcription factor engagement by PGC-1α than previously suggested. Second, the combination of PGC-1α ChIPseq and gene expression data revealed that of the high number of PGC-1α repressed genes, only a small minority, ~5%, have a PGC-1α DNA recruitment peak within a distance of ±10 kb of their promoter. Accordingly, the findings imply that the effect of PGC-1α on gene repression is predominantly indirect and that PGC-1α lacks an intrinsic inhibitory function (Baresic et al., 2014). While a systematic analysis of gene repression by PGC-1α remains to be done, PGC-1α-dependent reduction of the activating phosphorylation of the p65 subunit of the nuclear factor κB (NF-κB; Eisele et al., 2013) could account for at least some of the indirect inhibitory effect of PGC-1α on pro-inflammatory muscle gene expression (Eisele and Handschin, 2014). Third, this systematic study revealed novel insights into the mechanisms that ensure functional redundancy and complementation of PGC-1α-mediated transcriptional control. Principal component analysis of the transcription factor binding motifs within the regions of PGC-1α DNA recruitment implied an important role for the activator protein-1 (AP-1) transcription factor complex in PGC-1α-controlled gene expression (Baresic et al., 2014). AP-1 is a well-studied stress response gene in various cellular contexts, but has so far never been associated with PGC-1α function in muscle. Interestingly, the group of direct targets for AP-1 and PGC-1α was significantly enriched in genes associated with the cellular response to hypoxia, including several regulators of vascularization. This regulation complements the previously discovered control of the expression of the vascular endothelial growth factor (VEGF) by PGC-1α and ERRα (Arany et al., 2008). These findings imply that this seemingly redundant usage of different transcription factors to regulate the same biological program ensures adequate regulation of this critical process. Alternatively, the ability of PGC-1α to enhance the transcriptional activity of different partners might also indicate that PGC-1α is able to regulate the respective target genes in different cellular contexts, e.g., by binding to ERRα in a metabolically stressed muscle cell and acting together with AP-1 upon reduced physoxic conditions (Figure 2). Exercise triggers a hypoxic response including an activation of the hypoxia-inducible factor-1 (HIF-1) in muscle cells by a lowering of the relative tissue oxygen availability due to a dysbalance between oxygen consumption and supply, exacerbated by contraction-mediated constriction of blood vessels (Lindholm and Rundqvist, 2015). Together, the current data highlight the vast complexity of diverse mechanisms by which PGC-1α exerts a pleiotropic response in muscle cells.

## Conclusions and outlook

In essence, PGC-1α is a protein docking platform that on one side is recruited to transcription factors bound to their target gene promoters and enhancers, and on the other side interacts with components of the histone acetyltransferase (Puigserver et al., 1999), TRAP/DRIP/mediator (Wallberg et al., 2003), and SWI/SNF (Li et al., 2008) co-regulator protein complexes. Thereby, PGC-1α greatly boosts transcription even though PGC-1α lacks any discernable intrinsic enzymatic activity. Despite this ostensible simplicity in function, PGC-1α can control highly complex transcriptional programs in various tissues with a significant impact on organ plasticity. The ability of PGC-1α to integrate different signaling pathways through a multitude of PTMs, selective activation of alternative promoters and expression of transcript variants could provide a mechanistic explanation for the key regulatory function of PGC-1α in the regulation of tissue phenotypes. However, more studies will be required to obtain a better understanding and overview on the different aspects of the regulation of a co-activator-controlled transcriptional network, which not only is of high interest to understand the basic biology, but could also have a significant clinical impact. In a physiological and pathophysiological context, elevation of PGC-1α in muscle promotes a high endurance phenotype and ameliorates various muscle diseases with different etiologies, including Duchenne muscular dystrophy (Handschin et al., 2007c), denervation-induced fiber atrophy (Sandri et al., 2006), or sarcopenia (Wenz et al., 2014), respectively. To date, it is not clear which functions of muscle PGC-1α are responsible for such a broad therapeutic effect (Handschin, 2009). Similarly, pharmacological agents that robustly, specifically and safely elevate PGC-1α in skeletal muscle in the desired therapeutic window remain elusive (Svensson and Handschin, 2014). Finally, based on studies with muscle-specific PGC-1α transgenic animals that have an accelerated development of insulin resistance on a high fat diet (Choi et al., 2008), which can only be rectified by *bona fide* physical activity (Summermatter et al., 2013), the application of so-called “exercise mimetics,” compounds that elicit exercise-like effects in muscle and other tissues, might be problematic. Therefore, to design partial exercise mimetics or new compounds that specifically activate certain functions of PGC-1α, better knowledge about upstream regulators and downstream effects on the transcriptional network are needed. In particular, even though a strong correlation between muscle PGC-1α expression, exercise, and diseases states has been repeatedly documented in humans (e.g., see Silvennoinen et al., 2015), information about the regulation and function of human muscle PGC-1α so far remains largely descriptive. Until further insights are obtained, physical activity thus remains a cheap and effective way for the prevention and treatment of many chronic diseases (Handschin and Spiegelman, 2008), at least in exercise-tolerant patients.

### Conflict of interest statement

The authors declare that the research was conducted in the absence of any commercial or financial relationships that could be construed as a potential conflict of interest.
